# Clinicopathologic and molecular analysis of embryonal rhabdomyosarcoma of the genitourinary tract: evidence for a distinct DICER1-associated subgroup

**DOI:** 10.1038/s41379-021-00804-y

**Published:** 2021-04-12

**Authors:** Felix K. F. Kommoss, Damian Stichel, Jaume Mora, Manel Esteller, David T. W. Jones, Stefan M. Pfister, Eva Brack, Marco Wachtel, Peter Karl Bode, Hans-Peter Sinn, Dietmar Schmidt, Thomas Mentzel, Friedrich Kommoss, Felix Sahm, Andreas von Deimling, Christian Koelsche

**Affiliations:** 1 Institute of Pathology, Department of General Pathology, Heidelberg University Hospital, Heidelberg, Germany; 2 Soft-Tissue Sarcoma Research Group, German Cancer Research Center (DKFZ), Heidelberg, Germany; 3 Hopp Children’s Cancer Center (KiTZ), Heidelberg, Germany; 4 Department of Neuropathology, Institute of Pathology, Heidelberg University Hospital, Heidelberg, Germany; 5 Clinical Cooperation Unit Neuropathology, German Cancer Consortium (DKTK), German Cancer Research Center (DKFZ), Heidelberg, Germany; 6 Department of Pediatric Onco‑Hematology and Developmental Tumor Biology Laboratory, Hospital Sant Joan de Déu, Barcelona, Catalonia Spain; 7 Josep Carreras Leukaemia Research Institute (IJC), Barcelona, Catalonia Spain; 8 Centro de Investigacion Biomedica en Red Cancer (CIBERONC), Madrid, Spain; 9 Institucio Catalana de Recerca i Estudis Avançats (ICREA), Barcelona, Catalonia Spain; 10 Physiological Sciences Department, School of Medicine and Health Sciences, University of Barcelona (UB), Barcelona, Catalonia Spain; 11 Pediatric Glioma Research Group, German Cancer Consortium (DKTK) and German Cancer Research Center (DKFZ), Heidelberg, Germany; 12 Division of Pediatric Neurooncology, German Cancer Research Center (DKFZ), Heidelberg, Germany; 13 Department of Pediatric Hematology and Oncology, Heidelberg University Hospital, Heidelberg, Germany; 14 Division of Pediatric Hematology/Oncology, Department of Pediatrics, Inselspital, Bern University Hospital, Bern, Switzerland; 15 Department of Oncology and Children’s Research Center, University Children’s Hospital, Zurich, Switzerland; 16 Institute of Pathology and Molecular Pathology, University Hospital Zurich (USZ), Zurich, Switzerland; 17 MVZ für Histologie, Zytologie und molekulare Diagnostik Trier GmbH, Trier, Germany; 18 Dermatopathologie Bodensee, Friedrichshafen, Germany; 19 Institute of Pathology, Medizin Campus Bodensee, Friedrichshafen, Germany

**Keywords:** Sarcoma, Cancer epigenetics

## Abstract

Embryonal rhabdomyosarcoma (ERMS) of the uterus has recently been shown to frequently harbor *DICER1* mutations. Interestingly, only rare cases of extrauterine DICER1-associated ERMS, mostly located in the genitourinary tract, have been reported to date. Our goal was to study clinicopathologic and molecular profiles of *DICER1*-mutant (*DICER1*-mut) and *DICER1*-wild type (*DICER1*-wt) ERMS in a cohort of genitourinary tumors. We collected a cohort of 17 ERMS including nine uterine (four uterine corpus and five cervix), one vaginal, and seven urinary tract tumors. DNA sequencing revealed mutations of *DICER1* in 9/9 uterine ERMS. All other ERMS of our cohort were *DICER1*-wt. The median age at diagnosis of patients with *DICER1*-mut and *DICER1*-wt ERMS was 36 years and 5 years, respectively. Limited follow-up data (available for 15/17 patients) suggested that *DICER1*-mut ERMS might show a less aggressive clinical course than *DICER1*-wt ERMS. Histological features only observed in *DICER1*-mut ERMS were cartilaginous nodules (6/9 *DICER1*-mut ERMS), in one case accompanied by foci of ossification. Recurrent mutations identified in both *DICER1*-mut and *DICER1*-wt ERMS affected *KRAS, NRAS*, and *TP53*. Copy number analysis revealed similar structural variations with frequent gains on chromosomes 2, 3, and 8, independent of *DICER1* mutation status. Unsupervised hierarchical clustering of array-based whole-genome DNA methylation data of our study cohort together with an extended methylation data set including different RMS subtypes from genitourinary and extra-genitourinary locations (*n* = 102), revealed a distinct cluster for *DICER1*-mut ERMS. Such tumors clearly segregated from the clusters of *DICER1*-wt ERMS, alveolar RMS, and *MYOD1*-mutant spindle cell and sclerosing RMS. Only one tumor, previously diagnosed as ERMS arising in the maxilla of a 6-year-old boy clustered with *DICER1*-mut ERMS of the uterus. Subsequent sequencing analysis identified two *DICER1* mutations in the latter case. Our results suggest that *DICER1*-mut ERMS might qualify as a distinct subtype in future classifications of RMS.

## Introduction

Rhabdomyosarcoma (RMS) represents the most common soft tissue sarcoma of children and adolescents, accounting for almost 50% of all pediatric soft tissue sarcomas [1]. The current WHO classification of soft tissue tumors defines embryonal rhabdomyosarcoma (ERMS), alveolar rhabdomyosarcoma (ARMS), pleomorphic rhabdomyosarcoma, and spindle cell and sclerosing rhabdomyosarcoma (SRMS) as separate subtypes of RMS [2]. Beyond this classification, recent clinicopathological studies have identified novel molecular subtypes of RMS including two variants of SRMS harboring either *MYOD1* mutations or *VGLL2/NCOA2* gene fusions, as well as RMS with a predominantly epithelioid phenotype and *TFCP2* gene fusions [3].

ERMS represents the most common subtype, being most prevalent within the head and neck region, followed by the genitourinary tract [4]. In the genitourinary tract ERMS most frequently arises in the vagina and the urinary tract of infants [5]. ERMS of the uterine cervix and corpus are uncommon and are usually associated with a later age of onset [6, 7]. While ERMS mostly arises sporadically, it may also develop in the context of various familial tumor predisposition syndromes, such as Li-Fraumeni syndrome (*TP53*), neurofibromatosis type 1 (*NF1*), Noonan syndrome (multiple genes), and Costello syndrome (*HRAS*) [8–12]. Interestingly, uterine ERMS may develop in connection with the pleuropulmonary blastoma (PPB) familial tumor predisposition syndrome, which is characterized by germline mutations in *DICER1* (DICER1 syndrome) [6, 13]. Neoplasms associated with the DICER1 syndrome usually arise in young children and adolescents, and may—besides ERMS—include PPB, multinodular goiter, cystic nephroma, Sertoli–Leydig cell tumor of the ovary, and other rare tumor entities [14]. DICER1 is part of the ribonuclease III family and plays an important role in modulating gene expression at the posttranslational level through the processing of miRNA. Mutations in *DICER1* may thus contribute to tumorigenesis by activation of oncogenes through dysregulation of miRNA [15].

Recent studies have shown recurrent germline and somatic *DICER1* mutations to occur in a majority of uterine ERMS [7, 16, 17]. At the same time, only rare cases of extrauterine DICER1-associated ERMS, mostly located in the genitourinary tract, have been reported [16, 18–20]. In addition, molecular studies of larger cohorts of ERMS have failed to identify significant numbers of *DICER1* alterations, considering that most analyzed cases were located outside of the genitourinary tract [21–23]. Thus, the question arises, if *DICER1*-mutant (*DICER1*-mut) ERMS represents a separate clinicopathologic subgroup distinct from *DICER1*-wild type (*DICER1*-wt) ERMS.

In an attempt to contribute data to the ongoing discussion on *DICER1* alterations in ERMS, we report the clinicopathological characteristics of a cohort of genitourinary ERMS including *DICER1*-mut and *DICER1*-wt tumors by applying targeted DNA sequencing and performing comparative, genome-wide DNA methylation and copy number variation (CNV) analyses.

## Material and methods

### Study cohort and pathology review

A total of 17 ERMS of the genitourinary tract were collected, including nine uterine (four uterine cervix and five uterine corpus), one vaginal, and seven urinary-tract tumors (four bladders, one prostate, and two bladder/prostate not other specified). Uterine tumors were collected from the referral center archives of two of the authors (DSc, FK, *n* = 9). The remaining ERMS were allocated from the study archives of two of the authors (JM, ME, *n* = 3), as well as from the INFORM study cohort (*n* = 5) [24]. For all but one tumor, Hematoxylin & Eosin (H&E) slides from either fresh frozen (*n* = 2) or formalin-fixed and paraffin-embedded (FFPE) (*n* = 14) material was available. Additional desmin (Dako, mouse monoclonal, clone D33) and myogenin (Cell Marque, Rocklin, CA, USA, mouse monoclonal, clone F5D) immunohistochemistry (IHC) was only performed in cases were additional FFPE material was available (uterine tumors only, *n* = 9). All tumors with available H&E slides were subject to expert pathology review. A diagnosis of ERMS was made applying the current WHO criteria [2]. In cases of uterine tumors, special attention was given to the distinction from and the exclusion of uterine adenosarcoma, a well-known differential diagnosis of uterine ERMS [6]. Because this distinction is exceptionally difficult, for this study, uterine tumors where no consensus diagnosis of ERMS could be reached were excluded. Molecular features of two of the cases presented herein (ERMS 3 and 4) have previously been described elsewhere [25]. This study was approved by the institutional ethics committee and performed in accordance with the Declaration of Helsinki.

### DNA extraction

DNA was extracted from either fresh-frozen or FFPE tumor tissue. The Maxwell® 16 FFPE Plus LEV DNA Kit or the Maxwell® 16 Tissue DNA Purification Kit (for frozen tissue) was applied on the automated Maxwell device (Promega, Madison, WI, USA) according to the manufacturer’s instructions. A minimum of 100 ng DNA was extracted in every case and provided for subsequent DNA sequencing and array-based DNA methylation analysis.

### DNA sequencing

DNA was sequenced either as whole-exome using Agilent SureSelectXT Human V5 or V7 kit (*n* = 5, including from germline) or using a customized SureSelect XT technology (Agilent) panel (*n* = 14, no germline available) covering the coding regions of 130 genes. Library preparation, quality control, sequencing on a NextSeq or HiSeq sequencer (Illumina), and data processing were performed as previously described [24, 26]. Reads were aligned to the reference genome hg19 and variants were annotated using ANNOVAR software [27]. For samples without a matching germline control, synonymous and stop-loss variants, variants with a frequency exceeding 1% in the healthy population as well as variants described as known polymorphisms in the single nucleotide polymorphism database were excluded.

### Genome‑wide DNA methylation data generation, plotting and pre‑processing

DNA was also analyzed using the Illumina Infinium HumanMethylation450 (450 k) BeadChip or the EPIC/850k BeadChip (Illumina, San Diego, USA) at the Genomics and Proteomics Core Facility of the German Cancer Research Center (DKFZ) in Heidelberg. DNA methylation data were normalized by applying background correction and dye bias correction (shifting of negative control probe mean intensity to zero and scaling of normalization control probe mean intensity to 20,000, respectively). Probes targeting sex chromosomes, probes containing multiple single nucleotide polymorphisms, and probes that could not be uniquely mapped were removed.

### Copy number analysis, unsupervised clustering and t-SNE analysis

Copy number assessment was performed on methylation array data using the R-package conumee and copy number variants were identified by manual inspection [28]. For subsequent DNA methylation analyses, we included a previously compiled methylation data set of a large cohort of RMS of genitourinary and extra-genitourinary locations, including ARMS (*n* = 43), *MYOD1*-mutant SRMS (*n* = 12), as well as additional cases of ERMS (*n* = 39) and non-neoplastic striated muscle (control, *n* = 8), which has previously been published in part [29]. For unsupervised hierarchical clustering of DNA methylation data, 10,000 probes with the highest median absolute deviation across beta values were selected. Samples were hierarchically clustered using Euclidean distance and Ward’s linkage method. Methylation probes were reordered by hierarchical clustering using Euclidean distance and complete linkage. The unscaled methylation levels were shown in a heat map from the unmethylated state (blue color) to the methylated state (red color). For the unsupervised 2D representation of pairwise sample correlations, dimensionality reduction by t-distributed stochastic neighbor embedding (t-SNE) was performed using the 10,000 most variable probes, a perplexity of 10 and 3000 iterations. The stability of methylation groups was tested by varying the number of the most variable probes.

## Results

### *DICER1* mutations in ERMS of the genitourinary tract

**Fig. 1:**
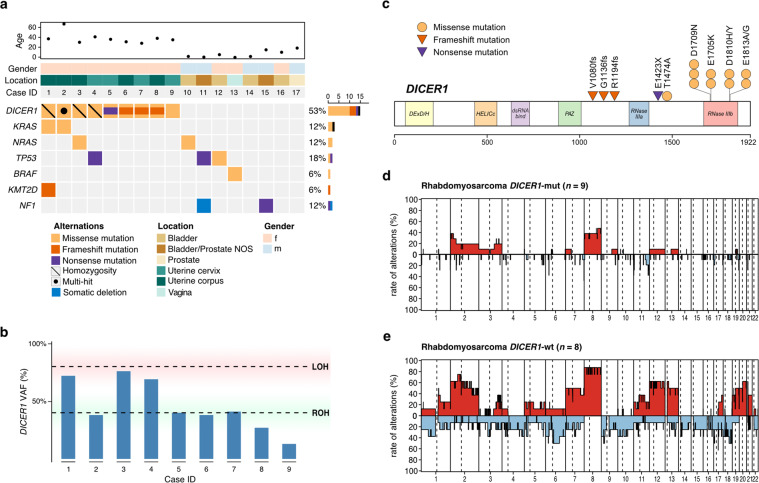
Clinical and molecular features of 17 ERMS of the genitourinary tract. **a** Age at diagnosis, gender distribution, sites of origin and DNA sequencing data, (**b**) variant allele frequency (VAF) of *DICER1* alterations suggesting either retained (ROH) or loss (LOH) of heterozygosity and (**c**) mutational spectrum of *DICER1* mutations in 9 uterine ERMS. Cumulative copy-number profiles of (**d**) 9 *DICER1*-mut ERMS and (**e**) 8 *DICER1*-wt ERMS. Molecular data of ERMS 3 and 4 have previously been reported elsewhere [25].

DNA sequencing of our cohort revealed a total of 14 *DICER1*-mutations in 9/9 (100%) uterine ERMS (Table 1, Fig. 1a, c). In detail, we identified ribonuclease III (RNase IIIb) domain hotspot mutations of *DICER1* in nine uterine ERMS. Four cases harbored additional nonsense or frameshift mutations of *DICER1*. In one case we identified an additional non-hotspot *DICER1* missense alteration (p.T1474A), which has previously been reported in hepatocellular carcinoma [30]. In four uterine ERMS, only the single RNase IIIb domain hotspot mutation of *DICER1* was identified. Analysis of the allelic fraction of the alteration indicated homozygous mutations in three of the latter tumors (ERMS 1, 3, and 4). Due to low tumor cell content in the fourth tumor (ERMS 9) the allelic fraction of the *DICER1* alteration was not informative of zygosity (Fig. 1b). Unfortunately, no germline data was available for *DICER1*-mut ERMS. No *DICER1* alteration was identified in any of the extrauterine genitourinary ERMS of our cohort (Fig. 1a).

**Table 1:** Clinicopathologic characteristics of 9 *DICER1*-mut and 8 *DICER1*-wt ERMS of the genitourinary tract. (*variant of unknown significance; NED = no evidence of disease, AWD = alive with disease, DOD = dead of disease).

Case ID	Gender	Age at primary Dx (years)	Site of origin	Histology					*DICER1* alterations		Clinical follow-up	
Case ID	Gender	Age at primary Dx (years)	Site of origin	Botryoid ERMS	Rhabdomyoblasts	Cartilage	Osteoid	Anaplasia	DNA transcripts	AA change	Follow-up (years)	Status
ERMS 1	f	37	Uterine Corpus	Yes	Yes	No	No	No	c.5113 G > A (Homozygous)	p.E1705K	9	NED
ERMS 2	f	67	Uterine Corpus	Yes	Yes	Yes	Yes	Yes	c.4420 A > G^a^ and c.5125 G > A	p.T1474A^a^ and p.D1709N	4	NED
ERMS 3	f	30	Uterine Corpus	No	No	Yes	No	No	c.5428 G > T (Homozygous)	p.D1810Y	7	NED
ERMS 4	f	41	Uterine Cervix	No	Yes	No	No	No	c.5125 G > A (Homozygous)	p.D1709N	8	NED
ERMS 5	f	36	Uterine Cervix	No	Yes	Yes	No	Yes	c.4267 G > T and c.5438 A > G	p.E1423X and p.E1813G	3	DOD
ERMS 6	f	31	Uterus Corpus	Yes	Yes	Yes	No	No	c.3238_3239insTGGCTT and c.5125 G > A	p.V1080fs and p.D1709N	NA	NA
ERMS 7	f	28	Uterine Cervix	Yes	Yes	Yes	No	No	c.3580delA and c.5113 G > A	p.R1194fs and p.E1705K	NA	NA
ERMS 8	f	38	Uterus Corpus	Yes	No	No	No	No	c.3405dupA and c.5428 G > C	p.G1136fs and p.D1810H	8	NED
ERMS 9	f	35	Uterus Cervix	Yes	No	Yes	No	No	c.5438 A > C	p.E1813A	9	NED
ERMS 10	m	3	Bladder	No	Yes	No	No	Yes	wt	–	1	AWD
ERMS 11	m	1	Bladder/ Prostate	No	Yes	No	No	No	wt	–	1	AWD
ERMS 12	f	5	Bladder	No	Yes	No	No	Yes	wt	–	15	NED
ERMS 13	f	0.5	Vagina	Yes	Yes	No	No	No	wt	–	11	DOD
ERMS 14	m	2	Bladder	Yes	Yes	No	No	No	wt	–	12	NED
ERMS 15	m	15	Bladder/Prostate	NA	NA	NA	NA	NA	wt	–	1	AWD
ERMS 16	f	10	Bladder	No	No	No	No	Yes	wt	–	7	AWD
ERMS 17	m	19	Prostate	No	Yes	No	No	No	wt	–	4	AWD

*NED* no evidence of disease, *AWD* alive with disease, *DOD* dead of disease.

^a^Variant of unknown significance.

### Clinicopathological features of *DICER1*-mut and *DICER1*-wt ERMS

**Fig. 2:**
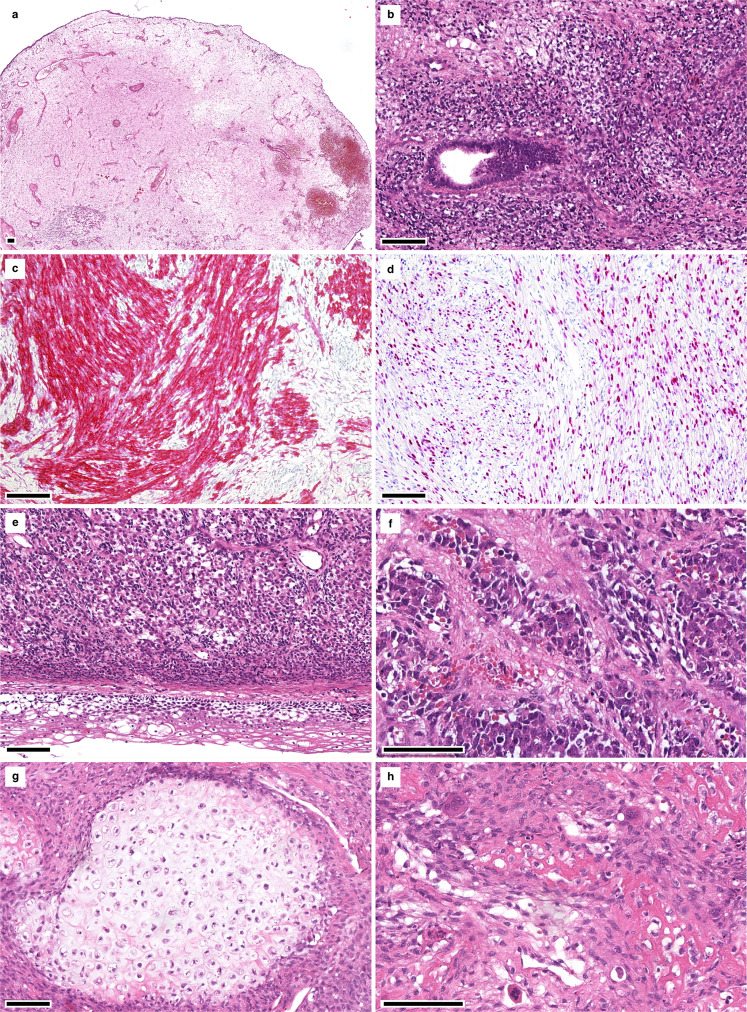
Histological features of *DICER1*-mut ERMS. **a** Nodular growth of hypocellular areas resembling the botryoid variant of ERMS, (**b**) entrapment of epithelium with cuffing of adjacent small blue cells with scant cytoplasm, as well as (**c**) desmin and (**d**) myogenin positivity. **e** Focal rhabdomyoblasts, and (**f**) anaplasia may be present. **g** Nodules of chondroid matrix as well as (**h**) areas of abutting ossification with osteoid matrix and multinucleated osteoclast-like giant cells may be suggestive of DICER1-association.

**Fig. 3:**
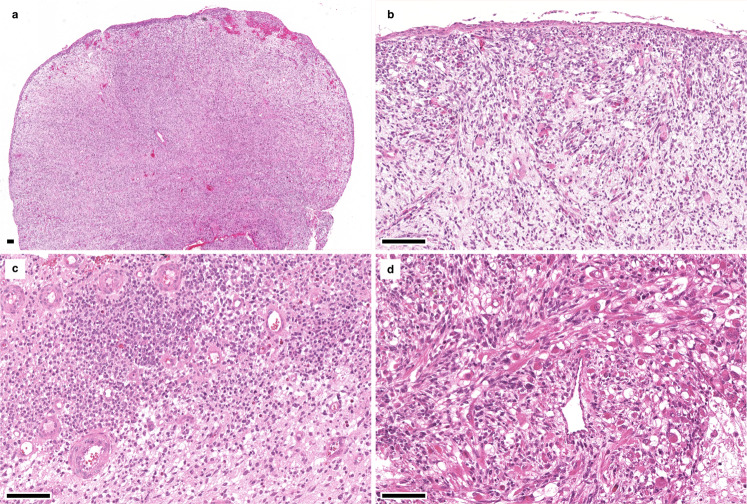
Histological features of *DICER1*-wt ERMS. **a** Botryoid variant of ERMS with (**b**) a distinct cambium layer and (**c**) proliferation of small blue cells with scant cytoplasm. **d** Anaplasia and rhabdomyoblasts may be present, however, in contrast to *DICER1*-mut ERMS no nodules of cartilage or osteoid are present.

Median patient age at primary diagnosis was 36 years for *DICER1*-mut ERMS (mean: 38.3 years, range: 28–67 years) and 5 years in cases of *DICER1*-wt ERMS (mean: 7 years, range: 0.5–19 years). While all *DICER1*-mut ERMS arose in women, the male-to-female ratio for *DICER1*-wt tumors was 1.6 (63% male and 37% female). Classical histomorphological features of ERMS were present in both *DICER1*-mut (Fig. 2) and *DICER1*-wt (Fig. 3) tumors. These included polypoid growth of hyper- and hypocellular areas of small blue cells with scant cytoplasm and varying foci of rhabdomyoblastic differentiation exhibiting tumor cells with abundant eosinophilic cytoplasm (so-called “strap cells”). Perivascular condensation of tumor cells was seen in hypocellular areas. A subset of ERMS exhibited polypoid exophytic growth with a cambium layer consisting of a linear subepithelial tumor cell condensation, resembling the botryoid variant of ERMS (sarcoma botryoides). Marked anaplasia in the form of poorly differentiated spindle cells with high-grade nuclear atypia was focally detected in *DICER1*-mut (1/9) and *DICER1*-wt (3/8) tumors. The majority of *DICER1*-mut ERMS (6/9) showed small foci of cartilaginous differentiation, while such differentiation did not occur in any of the *DICER1*-wt ERMS. Interestingly, one *DICER1*-mut ERMS (ERMS 2) arising in a 67-year-old patient showed focal ossification (formation of osteoid deposits with associated multinucleated osteoclast-like giant cells), abutting atypical nodular cartilaginous differentiation (Fig. 2a, b). IHC of *DICER1*-mut tumors showed immunoreactivity for desmin and at least focal positivity for myogenin in all *DICER1*-mut ERMS (*n* = 9). Limited clinical follow-up was obtained in 15/17 of selected cases. 6/7 *DICER1*-mut ERMS showed an uneventful clinical course (median follow-up time: 8 years, range: 3–9 years). The remaining patient died of disease after 3 years. Among *DICER1*-wt ERMS 7/9 patients of our series suffered tumor recurrences or died of disease (median follow-up time: 5.5 years, range: 1–15 years). Clinicopathological data are summarized in Table 1.

### Recurrent alterations affecting *TP53* and the RAS-pathway in *DICER1*-mut and *DICER1*-wt ERMS

Mutations identified in both *DICER1*-mut and *DICER1*-wt ERMS (Fig. 1a) affected *KRAS* and *NRAS* (3/9 *DICER1*-mut and 1/8 *DICER1*-wt ERMS) and *TP53* (1/9 *DICER1*-mut and 2/8 *DICER1*-wt ERMS). Alterations only identified in either *DICER1*-mut or *DICER1*-wt ERMS affected *KTM2D* (1/9 *DICER1*-mut ERMS), *BRAF* (1/8 *DICER1*-wt ERMS), and *NF1* (1/8 *DICER1*-wt ERMS). CNV identified in both *DICER1*-mut and *DICER1*-wt ERMS included frequent gains on chromosomes 2, 3, and 8 (Fig. 1d, e). While no amplifications were noted in any ERMS of our cohort, one *DICER1*-wt tumor harbored a somatic deletion including the *NF1* locus. Detailed DNA sequencing results are provided in Supplementary Table 1.

### Distinct patterns of DNA methylation in *DICER1*-mut ERMS

**Fig. 4:**
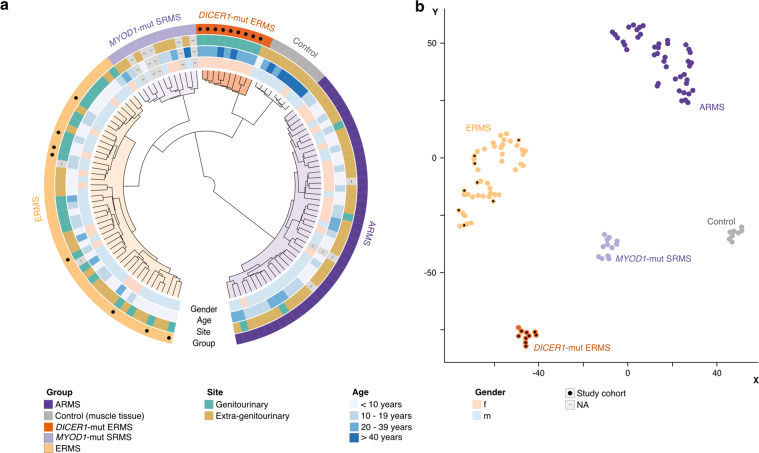
Distinct patterns of DNA methylation in *DICER1*-mut ERMS. Unsupervised hierarchical clustering (**a**) and t-SNE analysis (**b**) of 9 *DICER1*-mut and 8 *DICER1*-wt ERMS, together with a large methylation data set of RMS of genitourinary and extra-genitourinary locations shows distinct cluster formation for ARMS (*n* = 43), *MYOD1*-mut SRMS (*n* = 12), ERMS (*n* = 46), non-neoplastic striated muscle tissue (control; *n* = 8) and *DICER1*-mut ERMS (*n* = 10).

Unsupervised hierarchical clustering analysis of 17 ERMS of our cohort revealed two distinct methylation clusters corresponding to *DICER1*-mut and *DICER1*-wt ERMS (Supplementary Fig. 1a). Unsupervised hierarchical clustering (Fig. 4a) and t-SNE analysis (Fig. 4b) of 17 ERMS of our cohort, together with a large methylation data set of RMS of genitourinary and extra-genitourinary locations, confirmed a distinct *DICER1*-mut ERMS cluster. Such tumors clearly segregated from clusters of *DICER1*-wt ERMS, alveolar RMS, *MYOD1*-mutant spindle cell, and sclerosing RMS and non-neoplastic striated muscle samples. Methylation clusters remained stable when varying the analyzed number of CpG sites (data not shown). Interestingly, one ERMS from the reference set, arising in the maxillary region of a 6-year-old boy clustered with *DICER1*-mut ERMS. Histomorphological evaluation of this case revealed classical features of ERMS with focal anaplasia, however, no foci of cartilaginous differentiation were noted. Subsequent targeted DNA sequencing identified a missense (p.E844X) and an RNase IIIb domain hotspot mutation of *DICER1* (p.D1709N). The patient remains without evidence of disease 8 years after the initial diagnosis. Detailed information on the latter case is given in Supplementary Fig. 2.

## Discussion

Herein we present the clinicopathologic characteristics of a cohort of *DICER1*-mut and *DICER1*-wt ERMS of the genitourinary tract. We found *DICER1* alterations to be present in all ERMS of the uterine corpus and cervix, while no such mutations were identified in the remaining cases of our series. To date, only rare cases of *DICER1*-mut ERMS of the vagina and the urinary tract have been published [16, 31–33]. In keeping with the latter findings, most published series of molecularly analyzed ERMS arising in the vagina or urinary tract have lacked any tumors with *DICER1* mutations [16, 21, 22]. In contrast, most cases of molecularly analyzed ERMS of the uterine corpus and cervix harbor *DICER1* mutations [7]. In addition, few cases of ERMS arising in the broad ligament, the ovary, and the fallopian tube are also *DICER1* mutation-positive [18, 20]. In combination with published evidence, our results suggest that ERMS of the inner female genital tract—maybe with the exception of most tumors arising in the vagina—are associated with *DICER1* mutations, in contrast to other ERMS arising in the urinary tract which is usually *DICER1*-wt. In their recent paper, Appellaniz-Ruiz et al. hypothesized that *DICER1* mutations could predispose to tumor development in Müllerian-derived tissues [34]. The Müllerian ducts ontogenetically give rise to the upper portion of the vagina, the uterine cervix, and corpus, as well as the fallopian tubes, potentially providing a developmental explanation for the predilection of *DICER1*-mut ERMS at these sites.

Our results further indicate clinical differences between *DICER1*-mut and *DICER1*-wt ERMS. Here, the presence of *DICER1* alterations in ERMS was associated with older patient age at diagnosis (median age 36 years vs. 5 years for *DICER1*-wt ERMS). These results are in line with a recent report by de Kock et al. who reported a series of 19 DICER1-associated uterine ERMS with a median age at diagnosis of 30 years [7]. In contrast, a younger median age of 16 years at diagnosis of patients with DICER1-associated ERMS arising anywhere in the female genital tract was published in a recent meta-analysis [34]. Our results suggest that on the other hand most extrauterine ERMS, more specifically *DICER1*-wt ERMS, usually do not occur in older patients [5]. Although potentially limited by a selection bias, in our study, follow-up data suggest that in the genitourinary tract, *DICER1*-mut ERMS might show a more favorable clinical course as compared to *DICER1*-wt ERMS. Interestingly, genitourinary RMS arising in so-called non-bladder/non-prostate sites, such as the vagina or the uterus was reported to behave less aggressively than those with primary location in the urinary tract, irrespective of *DICER1* mutation status in two studies [35, 36]. While clinical information on DICER1-associated ERMS has rarely been published to date, potentially aggressive behavior of these tumors is exemplified by one of our patients who died of disease 3 years after initial diagnosis. Further, McCluggage et al. recently reported a *DICER1*-mut ERMS of the ovary, which relapsed only 3 months after surgery [20]. Given the above, further investigations of the clinical behavior of *DICER1*-mut ERMS are clearly needed.

Histologically, ERMS of the genitourinary tract involving epithelial-lined viscera such as the vagina, the bladder, and the uterus, often resemble botryoid ERMS [37–39]. Despite a similar or in some instances even identical morphology, the presence of cartilaginous nodules has previously been described in DICER1-associated ERMS, a finding that is confirmed by the current study [7, 20]. Similarly, we report osteoid formation in conjunction with *DICER1* mutations in a uterine ERMS, which has previously been reported in two cases of DICER1-associated ERMS of the female genital tract [34, 40]. Thus, identification of cartilaginous nodules and/or osteoid formation in ERMS of the genitourinary tract, including the uterus, may suggest a DICER1-association. Therefore, patients with this diagnosis should be referred to genetic counseling.

*DICER1*-wt ERMS are known to frequently harbor alterations impacting the RAS–RAF–MAPK (mitogen-activated protein kinase 1) pathway, including mutations in *RAS* genes (*NRAS*, *KRAS*, and *HRAS)* and *TP53* [21, 41]. Furthermore, genetic hallmarks of *DICER1*-wt ERMS may include aneuploidy with chromosome gains most frequently involving chromosomes 2, 3, and 8, as well as copy number neutral loss of heterozygosity (cnLOH) of chr11p alleles (11p15.5) [21, 42]. Interestingly, we identified similar patterns of aneuploidy as well as mutations affecting *TP53* and activating the RAS signaling cascade in both *DICER1*-mut and *DICER1*-wt ERMS. Preliminary analysis of single nucleotide polymorphism data from gene panel sequencing suggests that cnLOH of chr11p, typically a hallmark of ERMS, may not be a common feature of *DICER1*-mut ERMS. The panel data, however, do not cover sufficient loci in this region to be conclusive, and the 11p status in this subgroup will need confirming with more detailed analysis in the future.

A key finding of our study is that *DICER1*-mut and *DICER1*-wt ERMS are defined by distinct DNA methylation profiles, irrespective of their primary location. Recent studies have shown whole-genome DNA methylation profiling to be helpful in the identification of novel tumor entities and to be able to reliably assign CNS and mesenchymal tumors to diagnostic groups, including RMS and various uterine neoplasms [22, 29, 43–46]. The rationale behind this approach is the assumption that cancer cell-specific DNA methylation patterns to some extent recapitulate the DNA methylation patterns of their originating precursor cell, which are retained through cell division and tumorigenesis [47, 48]. Developmentally, RMS is believed to originate from myogenic progenitors, a concept supported by the expression of myogenic genes such as desmin and myogenin in RMS tumor cells [49–51]. Yet, studies have suggested that RMS may also develop from transdifferentiating non-myogenic cells, such as endothelial progenitors [52]. While the cell of origin of RMS remains elusive and may vary contextually, our results may suggest a differing cell lineage of *DICER1*-mut and *DICER1*-wt ERMS.

In DICER1 syndrome-associated neoplasms, predisposing loss-of-function mutations in *DICER1* typically occur together with a characteristic somatic missense hotspot mutation on the second allele [13]. However, also sporadic DICER1-associated neoplasms with biallelic *DICER1* alterations have been identified in the absence of germline alterations [53]. Although no germline DNA was available for study in the current paper, four *DICER1*-mut ERMS showed a combination of a *DICER1* frameshift or nonsense, and a *DICER1* hotspot mutation, consistent with alterations previously reported in DICER1 syndrome associated ERMS [7, 16]. The remaining DICER1-associated ERMS of our cohort showed multiple missense mutations or homozygous hotspot mutations in *DICER1*. Interestingly, the *DICER1* locus (14q) in the latter neoplasms of our cohort did not show any copy number alterations, particularly no deletion/microdeletion. However, we cannot rule out that a cnLOH may inactivate the second *DICER1* wild-type allele in these cases [54].

In the uterus, *DICER1* alterations are not limited to ERMS but have also been identified in a significant number of uterine adenosarcomas and few cases of carcinosarcoma [55, 56]. Adenosarcoma is a mesenchymal neoplasm typically exhibiting a phyllodes-like growth which may show significant morphological overlap with ERMS, especially when exhibiting sarcomatous overgrowth and/or rhabdomyoblastic differentiation [57]. A recent paper by de Kock et al. has elegantly shown that although *DICER1* alterations may be significantly more frequent in uterine ERMS, a distinction between uterine ERMS and adenosarcoma, based on *DICER1* mutations status alone is not always possible [7]. In keeping with this finding, few cases of uterine tumors resembling ERMS were excluded from our study after pathology review, as no consensus diagnosis could be reached due to equivocal histologic features, thus limiting the number of uterine cases in our series. Future studies investigating the potential of DNA methylation-based profiling in clarifying the relationship of the latter neoplasms are warranted.

Apart from uterine sarcomas, *DICER1* alterations have also been reported in other rare sarcoma entities, such as anaplastic sarcoma of the kidney and a recently described primary intracranial sarcoma with *DICER1* mutation [25, 58, 59]. Furthermore, *DICER1*-associated sarcomas of various locations show a significant morphological overlap with other DICER1-associated neoplasms such as PPB and Sertoli–Leydig-cell tumors [60, 61]. Shared histological features may include undifferentiated small blue cells, poorly differentiated spindle cells with areas of rhabdomyoblastic differentiation, a subepithelial cambium layer, chondroid differentiation as well as bone/osteoid formation [58]. Consequently, a novel unifying nomenclature for such neoplasms has been proposed [62]. However, more detailed comparative studies investigating the clinical and molecular characteristics of such rare *DICER1*-associated neoplasms arising in different anatomical locations are needed in order to clarify whether such tumors are in fact part of the same biological tumor spectrum.

Herein we describe the clinicopathologic features of a cohort of genitourinary ERMS in relation to *DICER1* mutation status. While all genitourinary ERMS showed overlapping morphological features, diverging clinicopathological characteristics and distinct DNA methylation profiles imply that *DICER1*-mut ERMS might qualify as a distinct subtype in future classifications of RMS.

## Supplementary information

Supplementary Material

## Data Availability

The data for this study data are available upon reasonable request.
