# Supplementary material for: Clinicopathologic and molecular analysis of embryonal rhabdomyosarcoma of the genitourinary tract: evidence for a distinct DICER1-associated subgroup
Source: Mod Pathol. 2021 Apr 12;34(8):1558–69. doi: 10.1038/s41379-021-00804-y (PMC8295035; doi:10.1038/s41379-021-00804-y)
Supplement: Supplementary file 1 — Supplementary Material [file 41379_2021_804_MOESM1_ESM.pdf]

Supplementary Figure 1: Unsupervised hierarchical clustering (**a**) of 9 *DICER1*-mut and 8 *DICER1*-wt ERMS, (**b**) together with a large methylation data set of RMS of genitourinary and extra-genitourinary locations shows distinct cluster formation for ARMS (n = 43), *MYOD1*-mut SRMS (n = 12), ERMS (n = 46), non-neoplastic striated muscle tissue (control; n = 8) and *DICER1*-mut ERMS (n = 10).

a

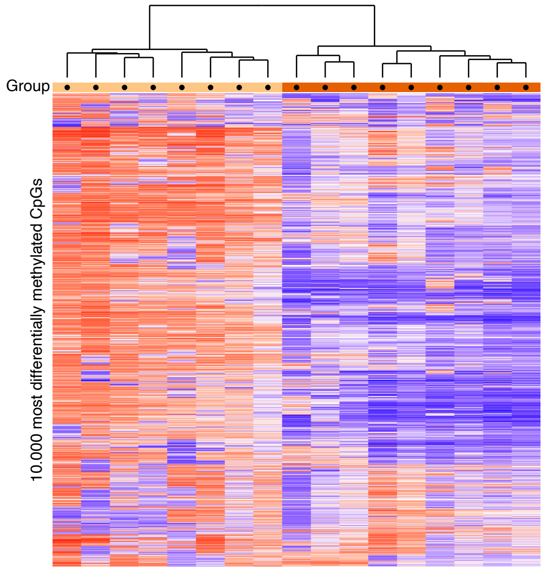

b

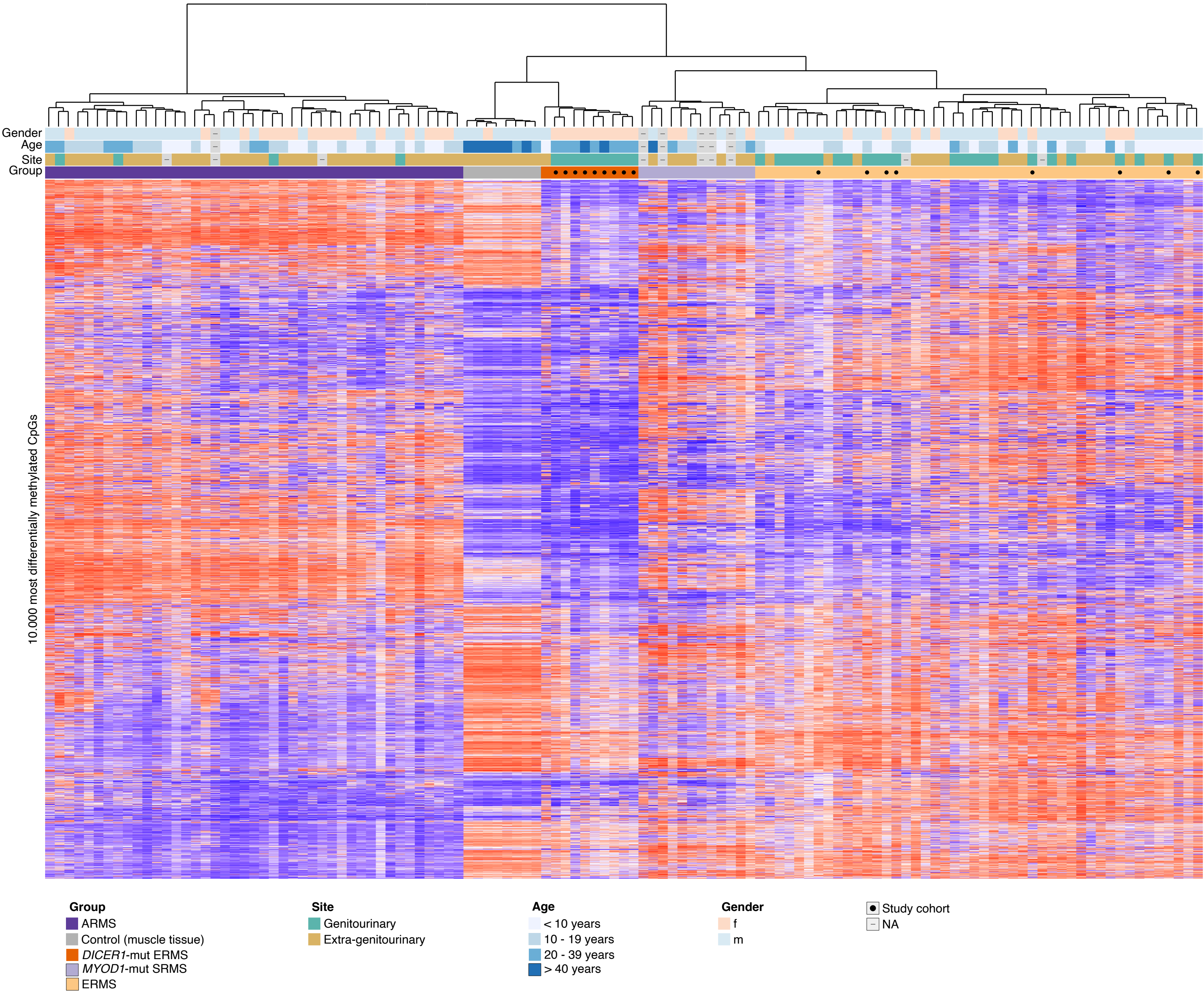

Supplementary Figure 2: ERMS arising in the maxillary region of a 6-year-old male which clustered with *DICER1*-mut ERMS of the genitourinary tract. (**a**) Histomorphological evaluation of this case revealed classical features of ERMS alongside foci of anaplasia. No foci of chondroid matrix were noted. (**b**) Subsequent targeted DNA sequencing identified a missense (p.E844X) and a RNase IIIb domain hotspot mutation of *DICER1* (p.D1709N).

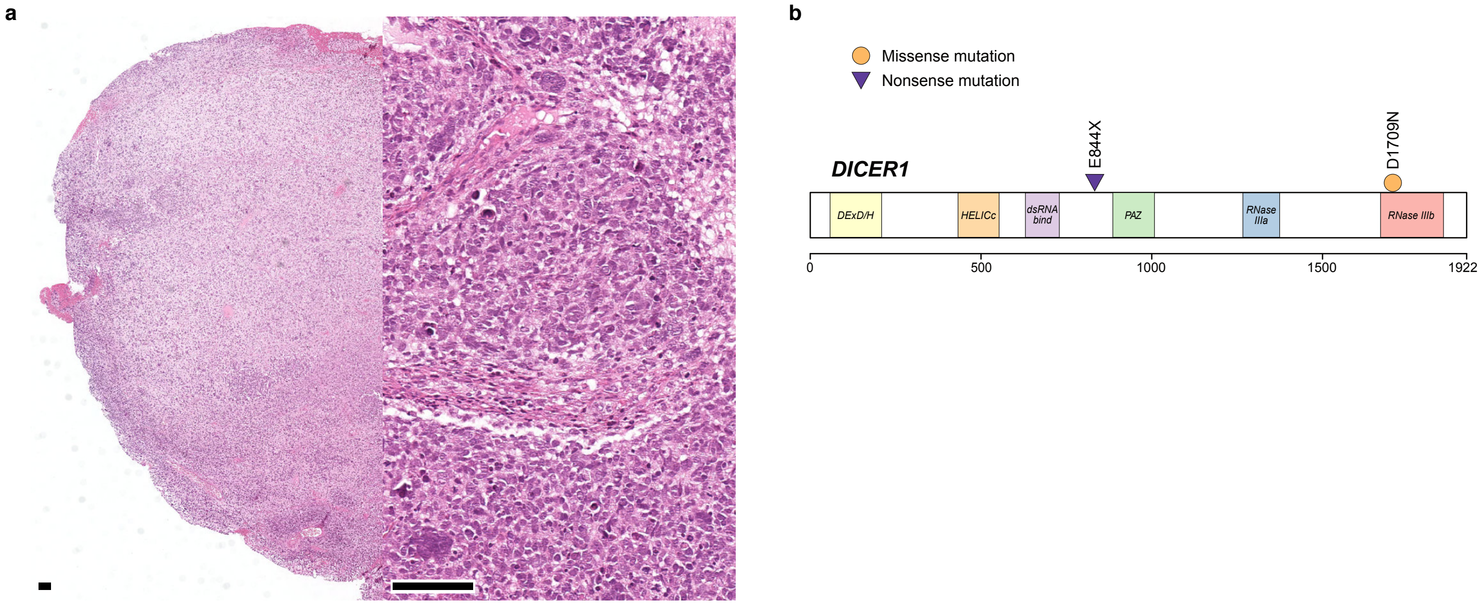

Supplementary Table 1: Variants called by next generation sequencing in 17 ERMS are depicted in a two-tier ranking of evidence separated in probably (red colored) clinically relevant and possible (blue colored) clinical relevance.

ID	Sequencing method	Material	Gene	AA Change	SNV Classification	Sequencing depth	VAF	Transcript	Mutation position	Chromosome
ERMS 1	PANEL	DNA_FFPE	CHD7	M2527L	nonsynonymous SNV	240	0,43	CHD7:NM_017780:exon34:c.A7579C:p.M2527L	61769418	8
ERMS 1	PANEL	DNA_FFPE	DICER1	E1705K	nonsynonymous SNV	221	0,87	DICER1:NM_001271282:exon24:c.G5113A:p.E1705K	95560476	14
ERMS 1	PANEL	DNA_FFPE	KMT2D	4470_4471del	frameshift deletion	400	0,18	KMT2D:NM_003482:exon39:c.13410_13411del:p.4470_4471del	49425077	12
ERMS 1	PANEL	DNA_FFPE	KRAS	Q61R	nonsynonymous DNV	323	0,39	KRAS:NM_033360:exon3:c.CA181_182GC:p.Q61R	25380277	12
ERMS 2	PANEL	DNA_FFPE	DICER1	T1474A	nonsynonymous SNV	176	0,34	DICER1:NM_001271282:exon23:c.A4420G:p.T1474A	95562837	14
ERMS 2	PANEL	DNA_FFPE	DICER1	D1709N	nonsynonymous SNV	208	0,39	DICER1:NM_001271282:exon24:c.G5125A:p.D1709N	95560464	14
ERMS 2	PANEL	DNA_FFPE	FGFR3	D758N	nonsynonymous SNV	324	0,36	FGFR3:NM_000142:exon17:c.G2272A:p.D758N	1808659	4
ERMS 2	PANEL	DNA_FFPE	IDH2	L385M	nonsynonymous SNV	272	0,32	IDH2:NM_002168:exon9:c.C1153A:p.L385M	90628258	15
ERMS 2	PANEL	DNA_FFPE	KRAS	G12V	nonsynonymous SNV	122	0,45	KRAS:NM_033360:exon2:c.G35T:p.G12V	25398284	12
ERMS 2	PANEL	DNA_FFPE	NDRG2	V51L	nonsynonymous SNV	78	0,49	NDRG2:NM_001282216:exon4:c.G151T:p.V51L	21490234	14
ERMS 3	PANEL	DNA_FFPE	DICER1	D1810Y	nonsynonymous SNV	126	0,77	DICER1:NM_001271282:exon25:c.G5428T:p.D1810Y	95557639	14
ERMS 3	PANEL	DNA_FFPE	NRAS	Y64D	nonsynonymous SNV	113	0,14	NRAS:NM_002524:exon3:c.T190G:p.Y64D	115256521	1
ERMS 3	PANEL	DNA_FFPE	TSC2	T573A	nonsynonymous SNV	294	0,42	TSC2:NM_001077183:exon17:c.A1717G:p.T573A	2120457	16
ERMS 4	PANEL	DNA_FFPE	DICER1	D1709N	nonsynonymous SNV	216	0,76	DICER1:NM_001271282:exon24:c.G5125A:p.D1709N	95560464	14
ERMS 4	PANEL	DNA_FFPE	MET	V136I	nonsynonymous SNV	190	0,51	MET:NM_001127500:exon2:c.G406A:p.V136I	116339544	7
ERMS 4	PANEL	DNA_FFPE	TP53	E48X	stopgain SNV	207	0,49	TP53:NM_001126116:exon1:c.G142T:p.E48X	7578392	17
ERMS 5	PANEL	DNA_FFPE	DICER1	E1423X	stopgain SNV	344	0,48	DICER1:NM_001271282:exon23:c.G4267T:p.E1423X	95562990	14
ERMS 5	PANEL	DNA_FFPE	DICER1	E1813G	nonsynonymous SNV	236	0,40	DICER1:NM_001271282:exon25:c.A5438G:p.E1813G	95557629	14
ERMS 6	PANEL	DNA_FFPE	APC	A59G	nonsynonymous SNV	159	0,40	APC:NM_000038:exon3:c.C176G:p.A59G	112102063	5
ERMS 6	PANEL	DNA_FFPE	CDKN2C	L47H	nonsynonymous SNV	169	0,39	CDKN2C:NM_001262:exon3:c.T140A:p.L47H	51439575	1
ERMS 6	PANEL	DNA_FFPE	DICER1	D1709N	nonsynonymous SNV	269	0,38	DICER1:NM_001271282:exon24:c.G5125A:p.D1709N	95560464	14
ERMS 6	PANEL	DNA_FFPE	DICER1	V1080fs	frameshift substitution	138	0,28	DICER1:NM_001271282:exon20:c.3238_3239TGGCTT	95571438	14
ERMS 6	PANEL	DNA_FFPE	KMT2C	Y987H	nonsynonymous SNV	323	0,38	KMT2C:NM_170606:exon18:c.T2959C:p.Y987H	151927025	7
ERMS 7	PANEL	DNA_FFPE	BRPF3	V1200I	nonsynonymous SNV	338	0,37	BRPF3:NM_015695:exon13:c.G3598A:p.V1200I	36198366	6
ERMS 7	PANEL	DNA_FFPE	DICER1	E1705K	nonsynonymous SNV	421	0,25	DICER1:NM_001271282:exon24:c.G5113A:p.E1705K	95560476	14
ERMS 7	PANEL	DNA_FFPE	DICER1	R1194fs	frameshift deletion	115	0,41	DICER1:NM_001271282:exon21:c.3580delA:p.R1194fs	95570153	14
ERMS 8	PANEL	DNA_FFPE	DICER1	D1810H	nonsynonymous SNV	498	0,29	DICER1:NM_001271282:exon25:c.G5428C:p.D1810H	95557639	14
ERMS 8	PANEL	DNA_FFPE	DICER1	G1136fs	frameshift insertion	509	0,26	DICER1:NM_001271282:exon21:c.3405dupA:p.G1136fs	95570327	14

ERMS 8	PANEL	DNA_FFPE	PTCH2	L462V	nonsynonymous SNV	856	0,33	PTCH2:NM_003738:exon11:c.T1384G:p.L462V	45294734	1
ERMS 8	PANEL	DNA_FFPE	TSC2	A1406V	nonsynonymous SNV	368	0,45	TSC2:NM_001114382:exon33:c.C4217T:p.A1406V	2134509	16
ERMS 8	PANEL	DNA_FFPE	TSC2	N1708K	nonsynonymous SNV	368	0,49	TSC2:NM_001114382:exon40:c.C5124A:p.N1708K	2138260	16
ERMS 9	PANEL	DNA_FFPE	DICER1	E1813A	nonsynonymous SNV	182	0,11	DICER1:NM_001271282:exon21:c.A5438C:p.E1813A	95557629	14
ERMS 9	PANEL	DNA_FFPE	KMT2C	Y816_I817delinsX	stopgain SNV	230	0,33	KMT2C:NM_170606:exon14:c.2447dupA:p.Y816_I817delinsX	151945071	7
ERMS 10	EXOME	DNA_FFPE	KMT2B	G1179R	nonsynonymous SNV	285	0,26	KMT2B:ENST00000301067.7:exon11:c.G3535C:p.G1179R	49443836	12
ERMS 10	EXOME	DNA_kryo	NRAS	Q61K	nonsynonymous SNV	110	0,54	NRAS:ENST00000369535.4:exon3:c.C181A:p.Q61K	115256530	1
ERMS 11	EXOME	DNA_kryo	TP53	R196X	stopgain	193	0,93	TP53:ENST00000359597.4:exon5:c.C586T:p.R196X	7578263	17
ERMS 12	PANEL	DNA_FFPE	GSE1	R763Q	nonsynonymous SNV	462	0,25	GSE1:NM_001278184:exon10:c.G2288A:p.R763Q	85697083	16
ERMS 12	PANEL	DNA_FFPE	KMT2C	Y987H	nonsynonymous SNV	333	0,33	KMT2C:NM_170606:exon18:c.T2959C:p.Y987H	151927025	7
ERMS 12	PANEL	DNA_FFPE	KMT2C	T316S	nonsynonymous SNV	396	0,61	KMT2C:NM_170606:exon7:c.A946T:p.T316S	151970856	7
ERMS 12	PANEL	DNA_FFPE	PDGFRA	N33Y	nonsynonymous SNV	470	0,25	PDGFRA:NM_006206:exon3:c.A97T:p.N33Y	55127309	4
ERMS 12	PANEL	DNA_FFPE	PIK3CG	972_981GCTGGTGGAC	nonframeshift substitution	1201	0,76	PIK3CG:NM_001282427:exon2:c.972_981GCTGGTGGAC	106508978	7
ERMS 12	PANEL	DNA_FFPE	TP53	R141C	nonsynonymous SNV	164	0,45	TP53:NM_001126116:exon4:c.C421T:p.R141C	7577121	17
ERMS 12	PANEL	DNA_FFPE	TSC2	1485_1486del	nonframeshift deletion	1142	0,21	TSC2:NM_001114382:exon34:c.4455_4457del:p.1485_1486del	2134982	16
ERMS 13	PANEL	DNA_FFPE	BRAF	V600E	nonsynonymous SNV	107	0,25	BRAF:NM_004333:exon15:c.T1799A:p.V600E	140453136	7
ERMS 13	PANEL	DNA_FFPE	PDGFRA	S851L	nonsynonymous SNV	113	0,28	PDGFRA:NM_006206:exon18:c.C2552T:p.S851L	55152120	4
ERMS 14	PANEL	DNA_FFPE	DDR1	P556L	nonsynonymous SNV	315	0,57	DDR1:NM_013994:exon12:c.C1667T:p.P556L	30864440	6
ERMS 14	PANEL	DNA_FFPE	KMT2C	Y816_I817delinsX	stopgain SNV	1190	0,21	KMT2C:NM_170606:exon14:c.2447dupA:p.Y816_I817delinsX	151945071	7
ERMS 14	PANEL	DNA_FFPE	PIK3CG	972_981GCTGGTGGAC	nonframeshift substitution	1148	0,66	PIK3CG:NM_001282427:exon2:c.972_981GCTGGTGGAC	106508978	7
ERMS 14	PANEL	DNA_FFPE	PLCH1	L1574R	nonsynonymous SNV	397	0,51	PLCH1:NM_001130960:exon23:c.T4721G:p.L1574R	155199118	3
ERMS 15	EXOME	DNA_kryo	NF1	Y1604X	stopgain	107	0,46	NF1:ENST00000356175.3:exon36:c.C4812G:p.Y1604X	29652877	17
ERMS 16	EXOME	DNA_kryo	ERBB2	E363K	nonsynonymous SNV	42	0,31	ERBB2:ENST00000269571.5:exon9:c.G1087A:p.E363K	37868640	17

Supplementary Table 2: DNA methylation data set including ARMS (n = 43), MYOD1-mutant SRMS (n = 12), ERMS (n = 39) and non-neoplastic striated muscle tissue (control; n = 8).

Diagnosis	Age group	Gender	SITE	Site	Previously reported
ARMS	10 - 19 years	m	extra genitourinary	Intracranial	Kölsche et al. (29)
ARMS	< 10 years	f	extra genitourinary	Lower extremity	-
ARMS	20 - 39 years	m	extra genitourinary	Lymph node	Kölsche et al. (29)
ARMS	< 10 years	f	NA	NA	Kölsche et al. (29)
ARMS	< 10 years	m	NA	NA	Kölsche et al. (29)
ARMS	NA	NA	NA	NA	Kölsche et al. (29)
ARMS	10 - 19 years	f	extra genitourinary	Soft tisse, trunk, thorax, paraspinal	Kölsche et al. (29)
ARMS	20 - 39 years	m	extra genitourinary	Soft tissue, head and neck, nasopharynx	Kölsche et al. (29)
ARMS	10 - 19 years	m	extra genitourinary	Soft tissue, head and neck, neck, lymph node	Kölsche et al. (29)
ARMS	10 - 19 years	m	extra genitourinary	Soft tissue, head and neck, tracheal region	Kölsche et al. (29)
ARMS	< 10 years	m	extra genitourinary	Soft tissue, limbs	Kölsche et al. (29)
ARMS	< 10 years	f	extra genitourinary	Soft tissue, limbs	Kölsche et al. (29)
ARMS	10 - 19 years	f	extra genitourinary	Soft tissue, limbs	Kölsche et al. (29)
ARMS	20 - 39 years	f	extra genitourinary	Soft tissue, limbs, lower limbs, foot	Kölsche et al. (29)
ARMS	< 10 years	f	extra genitourinary	Soft tissue, limbs, lower limbs, gluteal region	Kölsche et al. (29)
ARMS	< 10 years	f	extra genitourinary	Soft tissue, limbs, lower limbs, groin	Kölsche et al. (29)
ARMS	< 10 years	m	extra genitourinary	Soft tissue, limbs, lower limbs, groin, lymph node	Kölsche et al. (29)
ARMS	10 - 19 years	m	extra genitourinary	Soft tissue, limbs, lower limbs, leg	Kölsche et al. (29)
ARMS	< 10 years	f	extra genitourinary	Soft tissue, limbs, lower limbs, thigh	Kölsche et al. (29)
ARMS	10 - 19 years	f	extra genitourinary	Soft tissue, limbs, upper limbs, arm	Kölsche et al. (29)
ARMS	10 - 19 years	f	extra genitourinary	Soft tissue, limbs, upper limbs, axilla	Kölsche et al. (29)
ARMS	< 10 years	m	extra genitourinary	Soft tissue, limbs, upper limbs, hand	Kölsche et al. (29)
ARMS	10 - 19 years	m	extra genitourinary	Soft tissue, limbs, upper limbs, shoulder girdle, lymph node	Kölsche et al. (29)
ARMS	10 - 19 years	m	extra genitourinary	Soft tissue, trunk, abdomen, lymph node	Kölsche et al. (29)
ARMS	20 - 39 years	m	extra genitourinary	Soft tissue, trunk, abomen	Kölsche et al. (29)
ARMS	10 - 19 years	m	extra genitourinary	Soft tissue, trunk, buttock	Kölsche et al. (29)
ARMS	10 - 19 years	f	genitourinary	Soft tissue, trunk, pelvis, bladder wall	Kölsche et al. (29)
ARMS	10 - 19 years	m	genitourinary	Soft tissue, trunk, pelvis, genital	Kölsche et al. (29)
ARMS	20 - 39 years	m	genitourinary	Soft tissue, trunk, pelvis, genital	Kölsche et al. (29)
ARMS	20 - 39 years	m	genitourinary	Soft tissue, trunk, pelvis, genital	Kölsche et al. (29)
ARMS	< 10 years	f	extra genitourinary	Soft tissue, trunk, retroperitoneum	Kölsche et al. (29)
ARMS	< 10 years	m	extra genitourinary	Soft tissue, trunk, thoracic wall	Kölsche et al. (29)
ARMS	10 - 19 years	f	extra genitourinary	Soft tissue, trunk, thorax, mamma	Kölsche et al. (29)
ARMS	< 10 years	f	extra genitourinary	Soft tissue, trunk, thorax, mediastinum	Kölsche et al. (29)
ARMS	20 - 39 years	m	extra genitourinary	Visceral, thorax, lung	Kölsche et al. (29)
ARMS	10 - 19 years	m	extra genitourinary	Visceral, thorax, pleura	Kölsche et al. (29)
ARMS	10 - 19 years	f	extra genitourinary	Visceral, thorax, pleura	Kölsche et al. (29)
ARMS	10 - 19 years	m	extra genitourinary	Soft tissue, limbs, lower limbs, leg	-
ARMS	< 10 years	f	extra genitourinary	Soft tissue, trunk, abdomen	-
ARMS	10 - 19 years	m	extra genitourinary	Soft tissue, limbs, lower limbs	-
ARMS	< 10 years	m	extra genitourinary	Orbita	-
ARMS	< 10 years	m	extra genitourinary	Soft tissue, limbs, lower limbs, gluteal region	-
ARMS	< 10 years	m	extra genitourinary	Buccal	-
Control (striated muscle tissue)	> 40 years	m	extra genitourinary	Soft tissue, limbs	Kölsche et al. (29)
Control (striated muscle tissue)	> 40 years	m	extra genitourinary	Soft tissue, limbs, lower limbs, leg	Kölsche et al. (29)
Control (striated muscle tissue)	20 - 39 years	m	extra genitourinary	Soft tissue, limbs, lower limbs, thigh	Kölsche et al. (29)
Control (striated muscle tissue)	20 - 39 years	m	extra genitourinary	Soft tissue, limbs, lower limbs, thigh	Kölsche et al. (29)
Control (striated muscle tissue)	> 40 years	m	extra genitourinary	Soft tissue, limbs, lower limbs, thigh	Kölsche et al. (29)
Control (striated muscle tissue)	> 40 years	m	extra genitourinary	Soft tissue, limbs, lower limbs, thigh	Kölsche et al. (29)
Control (striated muscle tissue)	> 40 years	m	extra genitourinary	Soft tissue, limbs, upper limbs, forearm	Kölsche et al. (29)

Control (striated muscle tissue)	> 40 years	f	extra genitourinary	Soft tissue, limbs, upper limbs, forearm	Kölsche et al. (29)
ERMS (*DICER1* -mut)	< 10 years	m	extra genitourinary	Maxillary	-
ERMS	< 10 years	f	genitourinary	Bladder	-
ERMS	< 10 years	m	extra genitourinary	Intracranial	Kölsche et al. (29)
ERMS	< 10 years	m	NA	NA	Kölsche et al. (29)
ERMS	10 - 19 years	m	NA	NA	Kölsche et al. (29)
ERMS	< 10 years	m	extra genitourinary	Orbita	-
ERMS	< 10 years	m	extra genitourinary	Soft tissue, head and neck, oral cavity	Kölsche et al. (29)
ERMS	< 10 years	m	extra genitourinary	Soft tissue, head and neck, orbit	Kölsche et al. (29)
ERMS	10 - 19 years	m	extra genitourinary	Soft tissue, head and neck, orbit	Kölsche et al. (29)
ERMS	< 10 years	m	extra genitourinary	Soft tissue, trunk, abdomen	Kölsche et al. (29)
ERMS	< 10 years	f	extra genitourinary	Soft tissue, trunk, abdomen	Kölsche et al. (29)
ERMS	< 10 years	m	extra genitourinary	Soft tissue, trunk, abdomen	Kölsche et al. (29)
ERMS	20 - 39 years	f	extra genitourinary	Soft tissue, trunk, abdomen	Kölsche et al. (29)
ERMS	< 10 years	f	extra genitourinary	Soft tissue, trunk, abomen	Kölsche et al. (29)
ERMS	< 10 years	m	extra genitourinary	Soft tissue, trunk, pelvis	Kölsche et al. (29)
ERMS	20 - 39 years	f	extra genitourinary	Soft tissue, trunk, pelvis	Kölsche et al. (29)
ERMS	< 10 years	m	genitourinary	Soft tissue, trunk, pelvis, bladder wall	Kölsche et al. (29)
ERMS	10 - 19 years	m	genitourinary	Soft tissue, trunk, pelvis, genital	Kölsche et al. (29)
ERMS	10 - 19 years	m	genitourinary	Soft tissue, trunk, pelvis, genital	Kölsche et al. (29)
ERMS	10 - 19 years	m	genitourinary	Soft tissue, trunk, pelvis, genital	Kölsche et al. (29)
ERMS	10 - 19 years	m	genitourinary	Soft tissue, trunk, pelvis, genital	Kölsche et al. (29)
ERMS	10 - 19 years	m	genitourinary	Soft tissue, trunk, pelvis, genital	Kölsche et al. (29)
ERMS	10 - 19 years	m	genitourinary	Soft tissue, trunk, pelvis, genital	Kölsche et al. (29)
ERMS	20 - 39 years	m	genitourinary	Soft tissue, trunk, pelvis, genital	Kölsche et al. (29)
ERMS	20 - 39 years	m	genitourinary	Soft tissue, trunk, pelvis, genital	Kölsche et al. (29)
ERMS	< 10 years	m	extra genitourinary	Soft tissue, trunk, retroperitoneum	Kölsche et al. (29)
ERMS	< 10 years	m	extra genitourinary	Visceral, abdomen, small intestine	Kölsche et al. (29)
ERMS	10 - 19 years	m	extra genitourinary	Visceral, thorax, lung	Kölsche et al. (29)
ERMS	< 10 years	m	genitourinary	Prostate	-
ERMS	< 10 years	f	extra genitourinary	Orbita	-
ERMS	< 10 years	m	genitourinary	Paratesticular	-
ERMS	< 10 years	m	genitourinary	Prostate	-
ERMS	< 10 years	m	genitourinary	Prostate	-
ERMS	< 10 years	m	extra genitourinary	Parapharyngeal	-
ERMS	< 10 years	m	genitourinary	Paratesticular	-
ERMS	< 10 years	m	extra genitourinary	Orbita	-
ERMS	10 - 19 years	f	extra genitourinary	Ear canal	-
ERMS	< 10 years	f	genitourinary	Paravesicular	-
ERMS	< 10 years	m	extra genitourinary	Soft tissue, trunk, abdomen	-
MYOD1-mut SRMS	10 - 19 years	m	extra genitourinary	Bone, head and neck	Kölsche et al. (29)
MYOD1-mut SRMS	< 10 years	m	extra genitourinary	Bone, spine	Kölsche et al. (29)
MYOD1-mut SRMS	20 - 39 years	f	extra genitourinary	Intracranial, leptomeningeal	Kölsche et al. (29)
MYOD1-mut SRMS	20 - 39 years	f	extra genitourinary	Lymph node	Kölsche et al. (29)
MYOD1-mut SRMS	NA	NA	NA	NA	Kölsche et al. (29)
MYOD1-mut SRMS	NA	NA	NA	NA	Kölsche et al. (29)
MYOD1-mut SRMS	NA	NA	NA	NA	Kölsche et al. (29)
MYOD1-mut SRMS	NA	NA	NA	NA	Kölsche et al. (29)
MYOD1-mut SRMS	NA	NA	NA	NA	Kölsche et al. (29)
MYOD1-mut SRMS	> 40 years	m	extra genitourinary	Soft tissue, head and neck, head	Kölsche et al. (29)
MYOD1-mut SRMS	10 - 19 years	f	extra genitourinary	Soft tissue, head and neck, neck	Kölsche et al. (29)
MYOD1-mut SRMS	10 - 19 years	m	extra genitourinary	Soft tissue, trunk, thorax	Kölsche et al. (29)
